# An advanced *in vitro* human mucosal immune model to predict food sensitizing allergenicity risk: A proof of concept using ovalbumin as model allergen

**DOI:** 10.3389/fimmu.2022.1073034

**Published:** 2023-01-09

**Authors:** Marit Zuurveld, Cristina Bueno Díaz, Frank Redegeld, Gert Folkerts, Johan Garssen, Belinda van’t Land, Linette E.M. Willemsen

**Affiliations:** ^1^ Division of Pharmacology, Utrecht Institute for Pharmaceutical Sciences, Faculty of Science, Utrecht University, Utrecht, Netherlands; ^2^ Chemical Biology and Drug Discovery Group, Department of Pharmacological Sciences, Utrecht University, Utrecht, Netherlands; ^3^ Immunology Platform, Danone Nutricia Research B.V., Utrecht, Netherlands; ^4^ Center for Translational Immunology, University Medical Center Utrecht, Utrecht, Netherlands

**Keywords:** advanced *in vitro* model, allergic sensitization, food allergy, mucosal immunity, allergenicity risk assessment, ovalbumin

## Abstract

**Background:**

The global demand of sustainable food sources leads to introduction of novel foods on the market, which may pose a risk of inducing allergic sensitization. Currently there are no validated *in vitro* assays mimicking the human mucosal immune system to study sensitizing allergenicity risk of novel food proteins. The aim of this study was to introduce a series of sequential human epithelial and immune cell cocultures mimicking key immune events after exposure to the common food allergen ovalbumin from intestinal epithelial cell (IEC) activation up to mast cell degranulation.

**Methods:**

This *in vitro* human mucosal food sensitizing allergenicity model combines crosstalk between IEC and monocyte-derived dendritic cells (moDC), followed by coculture of the primed moDCs with allogenic naïve CD4+ T cells. During subsequent coculture of primed CD4+ T cells with naïve B cells, IgE isotype-switching was monitored and supernatants were added to primary human mast cells to investigate degranulation upon IgE crosslinking. Mediator secretion and surface marker expression of immune cells were determined.

**Results:**

Ovalbumin activates IEC and underlying moDCs, both resulting in downstream IgE isotype-switching. However, only direct exposure of moDCs to ovalbumin drives Th2 polarization and a humoral B cell response allowing for IgE mediated mast cell degranulation, IL13 and IL4 release in this sequential DC-T cell-B cell-mast cell model, indicating also an immunomodulatory role for IEC.

**Conclusion:**

This *in vitro* coculture model combines multiple key events involved in allergic sensitization from epithelial cell to mast cell, which can be applied to study the allergic mechanism and sensitizing capacity of proteins.

## 1 Introduction

The European Food Safety Authority (EFSA) updated their international guidelines in 2019 with new insights to assess adverse immune responses of novel food derived proteins (1). Food allergens may contain intrinsic properties to drive allergic sensitization, which can be designated as sensitizing allergenicity (2). Biological tools to predict adverse immune outcomes such as allergic sensitization are becoming increasingly relevant for safety assessment of new products, e.g. based on novel proteins before launching on the market due to the increasing number of individuals suffering from food allergies (3).

Key immunological events during food allergic sensitization have been identified (2). Allergic sensitization mainly starts at epithelial surfaces, upon contact with the allergenic food protein intestinal epithelial cells (IEC) become activated and start producing the type 2-driving alarmins IL25, IL33 and TSLP (4, 5). These epithelial-released proinflammatory mediators condition mucosal dendritic cells (DC) while sampling proteins in the Peyer’s patches (PP), gut lumen and/or lamina propria. Activated DCs can instruct naïve T and B cells in the PP or migrate to the mesenteric lymph nodes (MLN) for this purpose. Contact with an allergen and detection of local signals, including epithelial derived type 2 driving alarmins, promote the expression of MHC-II, costimulatory molecules and secretion of proinflammatory cytokines by DC (6). Allergen activated DCs are acting in the PP or MLN to drive T-helper 2 (Th2) cell polarization. By producing cytokines such as IL4 and IL13, Th2 cells promote immunoglobulin E (IgE) isotype-switching in allergen-specific B cells in the PP and/or MLN (7). *Via* the MLN the effector Th2 and B cells enter the blood stream and home back to the lamina propria (8). Within the mucosa, the Th2 cells are further activated and B cells differentiate into plasma cells, respectively secreting type 2 cytokines and allergen-specific IgE. Secreted IgE will bind to the FcϵRI receptor on mast cells and basophils in mucosal tissue, which sensitizes these cells for degranulation upon crosslinking of the FcϵRI-bound IgE by the allergen during a second exposure. Degranulation results in release of symptom-inducing mediators such as histamine, prostaglandins, mast cell proteases and proinflammatory type 2 cytokines (9).

In addition to IEC, many innate and adaptive immune cells, cellular molecules and humoral mediators involved in the allergic response have been studied extensively. However, due to the complexity of the gut-associated lymphoid tissue and the sequential organization of the pathological mechanism in food allergy development, it is difficult to determine the exact role and kinetics of individual cell types in a complete organism for sensitizing allergenicity studies. In addition, for ethical reasons it is essential to limit the use of animals for research purposes. Therefore, a growing body of research is focusing on the development of advanced *in vitro* mucosal immune models to allow effective safety and allergenicity risk assessment of novel food proteins and to provide tools for further mechanistic studies without the use of animal models (10). Recent attempts have aimed to develop sensitizing allergenicity models up till the T cell response (11, 12), however the use of murine cells may lack translational value to study food allergy from a human perspective. In a similar approach we recently showed the contribution of epithelial cells in driving the first steps of allergic sensitization (13). In the current manuscript, we also used the common food allergen ovalbumin, the most abundant protein in hen’s eggs (14), to develop a novel predictive and advanced *in vitro* human mucosal immune model. This model includes all major cell types involved in allergic sensitization and the allergic effector response, while allowing individual analysis of each single cell type. Therefore, this sequential mucosal food sensitizing allergenicity model facilitates further mechanistic studies and may be used as a first screening method to test intrinsic sensitizing capacities of novel food proteins.

## 2 Methods

### 2.1 Isolation and culture of cells

The human intestinal HT-29 cell line (passages 158-161) was cultured in McCoy’s 5A medium (Gibco, USA) containing 10% FCS (Gibco), 1% penicillin and streptomycin (pen/strep) (Sigma-Aldrich, UK). PBMCs were isolated from buffy coats from healthy donors, who gave consent that their donations could be used for research purposes (Dutch Blood Bank, The Netherlands), by density-gradient centrifugation in Leucosep tubes (Greiner). Subsequent isolation of monocytes, naïve T cells and naïve B cells was performed by negative selection and isolation of CD34+ stem cells by positive selection using appropriate magnetic separation kits according to the manufacturer’s protocol (Miltenyi Biotec, Germany). Monocytes from 3 independent donors were cultured for 6 days in RPMI 1640 (Lonza, Switzerland) containing 10% FCS, 1% pen/strep, 100ng/ml IL4 and 60ng/ml GM-CSF (Prospec, Israël) to allow differentiation into immature monocyte-derived dendritic cells (moDCs). Naïve T and naïve B cells were isolated from 3 independent donors, therefore naïve B cells were stored at –80°C in FCS containing 10% DMSO until further use. Both naïve T and naïve B cells were cultured in IMDM (Sigma-Aldrich) containing 5% FCS, 1% pen/strep, 20μg/ml apo-transferrin (Sigma-Aldrich) and 50μM β-mercaptoethanol (Sigma-Aldrich). Primary human mast cells were differentiated from CD34+ stem cells (15). Purity of isolated monocytes, naïve T and B cells was assessed immediately after isolation, see **Supplemental Figure 1**.

### 2.2 Sequential mucosal food sensitizing allergenicity model

**Figure 1:**
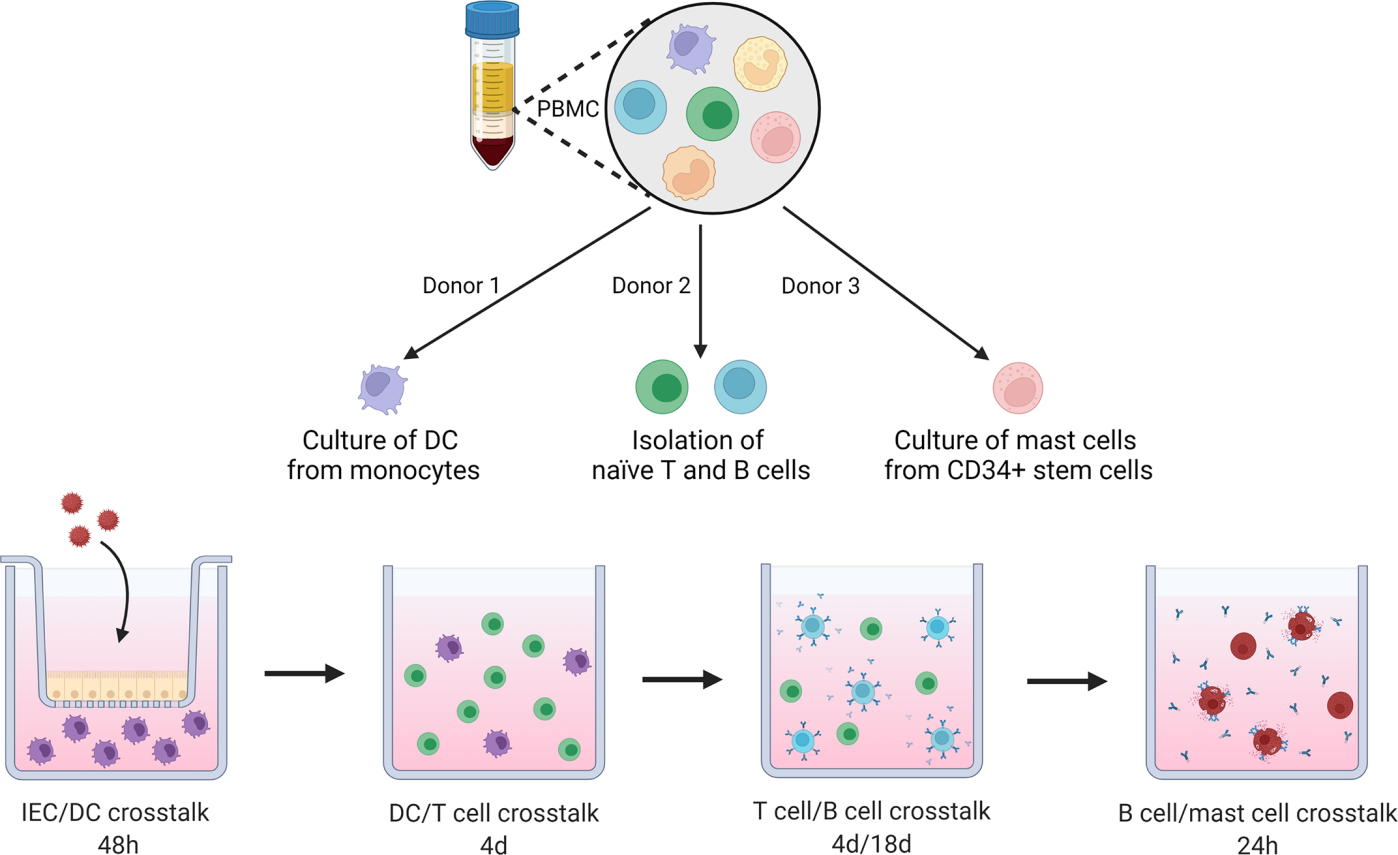
Schematic overview of coculture steps in this novel sequential mucosal food sensitizing allergenicity model. PBMCs are isolated from 3 donors, from the first donor, moDC are cultured from isolated monocytes. The second donor provides naïve T and B cells. The third donor is used to isolate CD34+ stem cells which are differentiated into primary human mast cells. moDC are exposed to OVA in presence or absence of IEC for 48h, primed moDCs are collected and cocultured with naïve Th cells in a 1:10 ratio for 4 days. Next, the primed Th cells are cocultured with naïve B cells for 4 and 18 days. The B cell activation status was assessed after 4 days. After 18 days of coculture supernatant was collected to determine antibody secretion and for incubation with primary human mast cells overnight. IgE specific mast cell degranulation was measured as well as cytokine secretion during a final 18h incubation of the primed mast cells. This figure was created with BioRender.com.

This advanced sequential *in vitro* coculture model was used to mimic cross talk in natural order of occurrence between relevant cell types in food allergic responses. A schematic overview of coculture steps is displayed in **Figure 1**.

HT-29 cells were 5 times diluted based on surface area and seeded in transwell inserts (polyester membrane, 0.4μm pores) (Corning Incorporated, USA). After 6 days HT29 cells reached confluency and 5x10E5 immature moDCs (in 1,5mL) were added to the basolateral compartment, alternatively moDCs were added to wells without IEC. OVA (100μg/mL (Sigma-Aldrich)) was added apically (in 0,5mL) to the IEC or empty transwell filter membranes for 48 hours.

Afterwards, viability of IEC was not affected as measured by a WST-assay to determine mitochondrial activity (**Supplemental Figure 2**) moDCs were collected for phenotyping by flow cytometry and coculture with allogenic naïve T helper cells (10:1 ratio (T cell:moDC)) in a 24 well flat-bottom plate for 4 days in the presence of 5ng/mL IL2 (Prospec) and 150ng/mL anti-CD3 (clone CLB-T3/2, Sanquin, The Netherlands), to allow generic T cell activation guided by the primed moDCs.

Following the moDC/T cell coculture, cells were collected again for phenotyping and cocultured with autologous (to T cells) 2x10E5 naïve B cells (1:1 ratio) in a 24 well flat-bottom plate in the presence of 5μg/mL anti-IgM (Sigma-Aldrich), to allow generic B cell activation guided by the primed T cells.

The activation status of the B cells was assessed after 4 days by flow cytometry. Cell free supernatant was collected after 18 days and added to primary human mast cells in a 1:1 dilution with fresh mast cell culture medium. After 24h incubation the supernatant was washed away and IgE-mediated degranulation (at 1 hour) and type 2 cytokine production (after 18h) by mast cells were determined. Appropriate control conditions for each step of the model are shown in **Supplemental Figure 3**.

### 2.3 Enzyme-linked immunosorbent assay (ELISA)

Supernatants collected from IEC, IEC/moDC, moDC, moDC/T cell, T cell/B cell and mast cell cultures were analyzed for cytokine, chemokine and immunoglobulin secretion. Concentrations of IFNγ, IL4, IL8, IL10, IL12p70, IL13, IL17, IgE, IgG, TGFβ, TSLP (Invitrogen, USA), IL15 (Biolegend, USA), CCL20, CCL22, IL25, IL33 (R&D systems, USA) were measured according to manufacturer’s instruction.

### 2.4 FACS

Phenotype of moDC, T cells and B cells after coculture was analyzed by flow cytometry. Collected cells were stained with Fixable Viability Dye 780-APC Cyanine 7 (eBioscience, USA), followed by blocking of nonspecific binding sites with human Fc block (BD Biosciences, USA) in PBS containing 1% bovine serum albumin (Roche, Switzerland). Extracellular staining was performed using titrated volumes of the following antibodies: CD11c-PerCP eFluor 710 (clone 3.9), HLA-DR-PE (clone LN3), CD80-FITC (clone 2D10.4), CD86-PE-Cy7 (clone IT2.2), OX40L-APC (clone RM134L), CD4-PerCP-Cy5.5 (clone OKT4), CXCR3-AF488 (clone 1C6/CXCR3), CRTH2-APC (clone BM16), CD19-PE-Cy7 (HIB19), CD4-PE (clone RPA-T4), CD25-AF488 (clone BC96) (purchased from eBioscience or BD Biosciences). Cells were permeabilized with the Intracellular Fixation & Permeabilization Buffer Set (eBioscience, USA) to allow staining with IL13-PE (clone JES10-5A2). Flow cytometric measurements were performed using BD FACS CantoII (Becton Dickinson, USA) and data was analyzed using FlowLogic software, (Inivai Technologies, Australia). Representative gating strategies are given in **Supplemental Figure 4**.

### 2.5 β-Hexosaminidase assays

After overnight incubation with B cell supernatant, mast cells were washed and incubated with mouse anti-human IgE (eBioscience) for 1 hour. Next, 158μM 4-methylumbelliferyl-β-d-glucopyranoside (4-MUG) was added to the cell-free supernatant for 1 hour. Enzymatic reaction was stopped with 0,1M glycine buffer (pH 7.8). 4-Methylumberriferone was quantified by measuring fluorescence at ex350nm/em460nm with a GloMax^®^ Discover Microplate Reader (Promega, USA). The percentage of β-hexosaminidase release was calculated as percentage relative to a positive control (100% degranulation) (Triton X-100) and negative control (0% degranulation). Mast cells were washed and fresh medium was added, mast cells were incubated for an additional 18h to measure secretion of cytokines.

### 2.6 Statistical analysis

Statistical analyses were performed using GraphPad Prism Version 9.4.1. Data was analyzed by paired t-test. p < 0.05 is considered statistically significant, and data is represented as mean ± SEM of n=3 independent repeats per dataset.

## 3 Results

### 3.1 Ovalbumin induces increased maturation in moDCs in absence of IEC

At first a food allergen encounters the intestinal epithelial barrier, therefore *in vitro* activation of IEC by the second most common food allergen ovalbumin (OVA) was explored by exposing IEC in a flat-bottom plate for 48h to different doses of OVA to determine optimal concentration for epithelial activation (**Supplemental Figure 5**). Exposure to 100 μg/mL OVA resulted in the significant secretion of IL8 and CCL20, therefore this concentration was used in following experiments.

**Figure 2:**
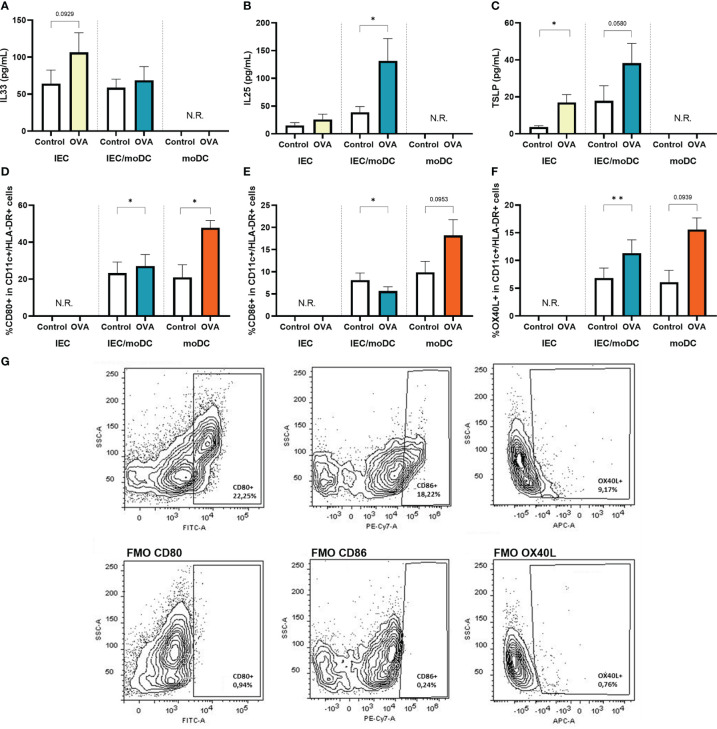
After 48h OVA exposure of IEC, IEC-moDC or moDC, secretion of epithelial derived alarmins **(A)** IL33, **(B)** IL25, and **(C)** TSLP was determined. In addition, the percentage of **(D)** CD80+, **(E)** CD86+ and **(F)** OX40L+ expressing moDCs (defined as CD11c+ and HLA-DR+) was measured. A representative sample and corresponding FMO controls are shown in **(G)**. Data is analyzed by paired t-test, n=3, mean ± SEM (* p<0.05, ** p<0.01).

Activation of IEC and/or moDCs in transwells by apically administered OVA was assessed by cytokine secretion and expression of costimulatory markers. Apical exposure of IEC in the transwell to OVA resulted in an enhanced basolateral secretion of IL33 and TSLP (**Figures 2A, C**). When IEC were exposed to OVA in presence of moDCs (OVA-IEC-DC), secretion of IL25 and TSLP was increased (**Figure 2B**). These epithelium derived cytokines were not measured in cultures with only moDCs. Coculture of IEC with moDCs (IEC-DC) resulted in an increased percentage of moDCs expressing CD80 and OX40L in response to OVA (**Figures 2D, F**), while exposing moDCs to OVA in absence of IEC (OVA-DC) tended to increase the frequency of moDCs expressing all maturation related costimulatory molecules (**Figures 2D–F**). Representative FACS plots including FMOs are shown in **Figure 2G**. Appropriate control conditions are shown in **Supplemental Figure 3**.

### 3.2 OVA enhances secretion of CCL20, CCL22 and IL8 irrespective of IEC presence

**Figure 3:**
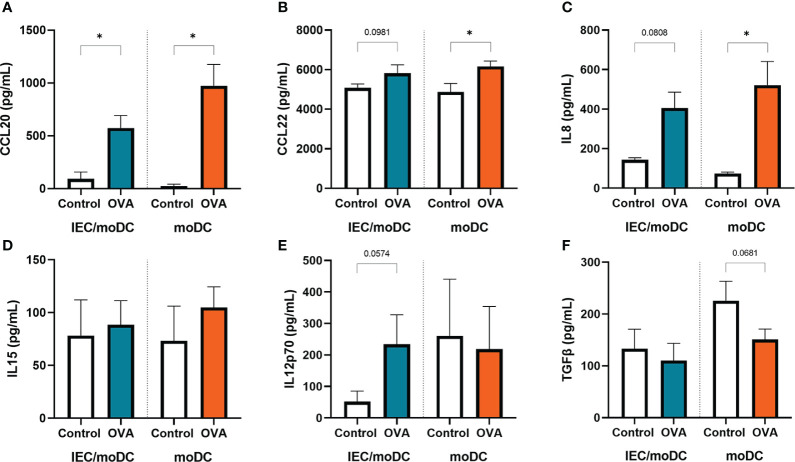
After 48h OVA exposure of IEC-moDC or moDC alone, secretion of **(A)** CCL20, **(B)** CCL22, **(C)** IL8, **(D)** IL15, **(E)** IL12p70, and **(F)** TGFβ was determined in the basolateral compartment. Data is analyzed by paired t-test, n=3, mean ± SEM (* p<0.05).

Cytokines and chemokines produced by IEC and/or moDCs during OVA exposure were measured. Both in presence or absence of IEC, secretion of CCL20, CCL22 and IL8 was increased (**Figures 3A–C**). IL15 (**Figure 3D**) secretion was not significantly affected during OVA exposure, while OVA-IEC-DC showed an inclining trend for IL12p70 (**Figure 3E**) and OVA-DC tended to decrease TGFβ levels (**Figure 3F**).

### 3.3 Type 2 mediators are increased in T cells after coculture with OVA-DC

**Figure 4:**
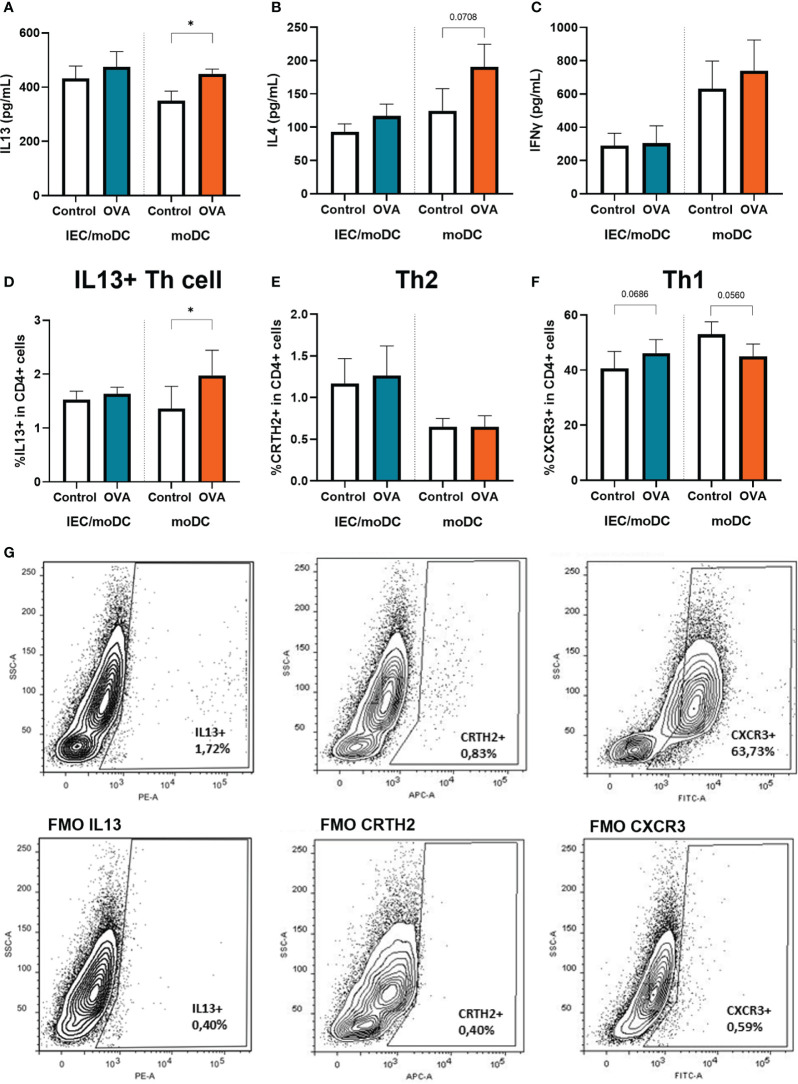
OVA-IEC-moDC or OVA-moDC were coculture with allogenic naïve T cells for 4 days. After the coculture period, secreted **(A)** IL13, **(B)** IL4, **(C)** IFNγ were measured in the basolateral supernatant. Furthermore, cells were analyzed by flow cytometry to determine the percentage of CD4+ cells expressing **(D)** IL13, **(E)** CRTH2, and **(F)** CXCR3. A representative sample and corresponding FMO controls are displayed in **G**). Data is analyzed by paired t-test, n=3, mean ± SEM (* p<0.05).

To investigate the immunological function of the OVA or OVA-IEC exposed moDCs, cells were cocultured for 4 days with allogenic naïve Th cells. The T cells were exposed to anti-CD3 and IL2 to allow a generic TCR activation, to be guided by the primed moDC. Secretion of IL13 and IL4, and the percentage of Th2 cells containing IL13 (**Figures 4A, B, D, G**) was increased in Th cells that were cocultured with OVA-DC compared to unexposed DC. This increase was not observed upon coculture with OVA-IEC-DC. The percentage of Th2 cells (CRTH2+) was not affected (**Figures 4E, G**), but the percentage of Th1 cells (CXCR3+) tended to increase when T cells were coculture with OVA-IEC-DC, while a decreasing trend was observed upon coculture with OVA-DC (**Figures 4F, G**). Th1 type IFNγ (**Figure 4C**) and Th17 type IL17 (data not shown) secretion remained unaffected. While Th cells primed with OVA-DC showed increased type 2 cytokine release, levels of regulatory IL10 were increased after coculture OVA-IEC-DC with T cells (data not shown).

### 3.4 Both OVA-DC-T cells and OVA-IEC-DC-T cells induce IgE secretion in B cells

**Figure 5:**
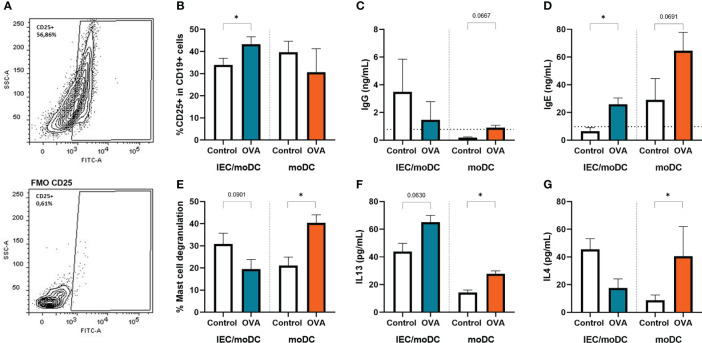
After the T cells were cocultured with OVA primed IEC-moDC or OVA primed moDC, subsequently the primed T-cells were cocultured with autologous naïve B cells. Activation of B cells was assessed at day 4 by flow cytometric analysis of expression of CD25, **(A)** a representative sample and appropriate FMO control are shown. **(B)** Percentage of CD25 expressing B cells are displayed for the used conditions. Secretion of **(C)** IgG and **(D)** IgE was determined in the supernatant after 18 days of coculture. These cell free 18 day supernatants were also overnight incubated with primary human mast cells. **(E)** Degranulation upon crosslinking with anti-IgE was determined by means of beta-hexosaminidase release as well as overnight **(F)** IL13, and **(G)** IL4 secretion by the primed and degranulated mast cells. Data is analyzed by paired t-test, n=3, mean ± SEM (* p<0.05).

To study whether DC-instructed Th cells were capable of inducing immunoglobulin production by B cells, OVA-DC and OVA-IEC-DC cocultured Th cells (OVA-DC-T and OVA-IEC-DC-T respectively) were incubated with autologous naïve B cells for 18 days. The B cells were stimulated with anti-IgM to allow a generic BCR activation, to be guided by the primed T cells. The activation status of the B cells was determined after 4 days, immunoglobulin secretion was measured after 18 days. OVA-IEC-DC-T cell coculture with naïve B cells (OVA-IEC-DC-T-B) increased the percentage of CD25+ activated B cells and IgE secretion (**Figures 5A, C**). OVA-DC-T cells did not induce CD25 expression in B cells (OVA-DC-T-B), but tended to enhance IgG and IgE secretion (**Figures 5B, C**). Subsequently, mast cells were primed with supernatant from OVA-IEC-DC-T-B and OVA-DC-T-B collected after 18 days of T/B cell coculture. Degranulation upon crosslinking with anti-IgE as indicated by % β-hexosaminidase release was measured and, after mast cells were kept in culture for another 18h, IL-13 and IL4 secretion were quantified. The IgE-mediated mast cell degranulation, as well as IL-13 and IL4 secretion were significantly increased when mast cells were primed with the OVA-DC-T-B supernatant, but not with the OVA-IEC-DC-T-B supernatant compared to their respective controls (**Figures 5D–G**).

## 4 Discussion

**Figure 6:**
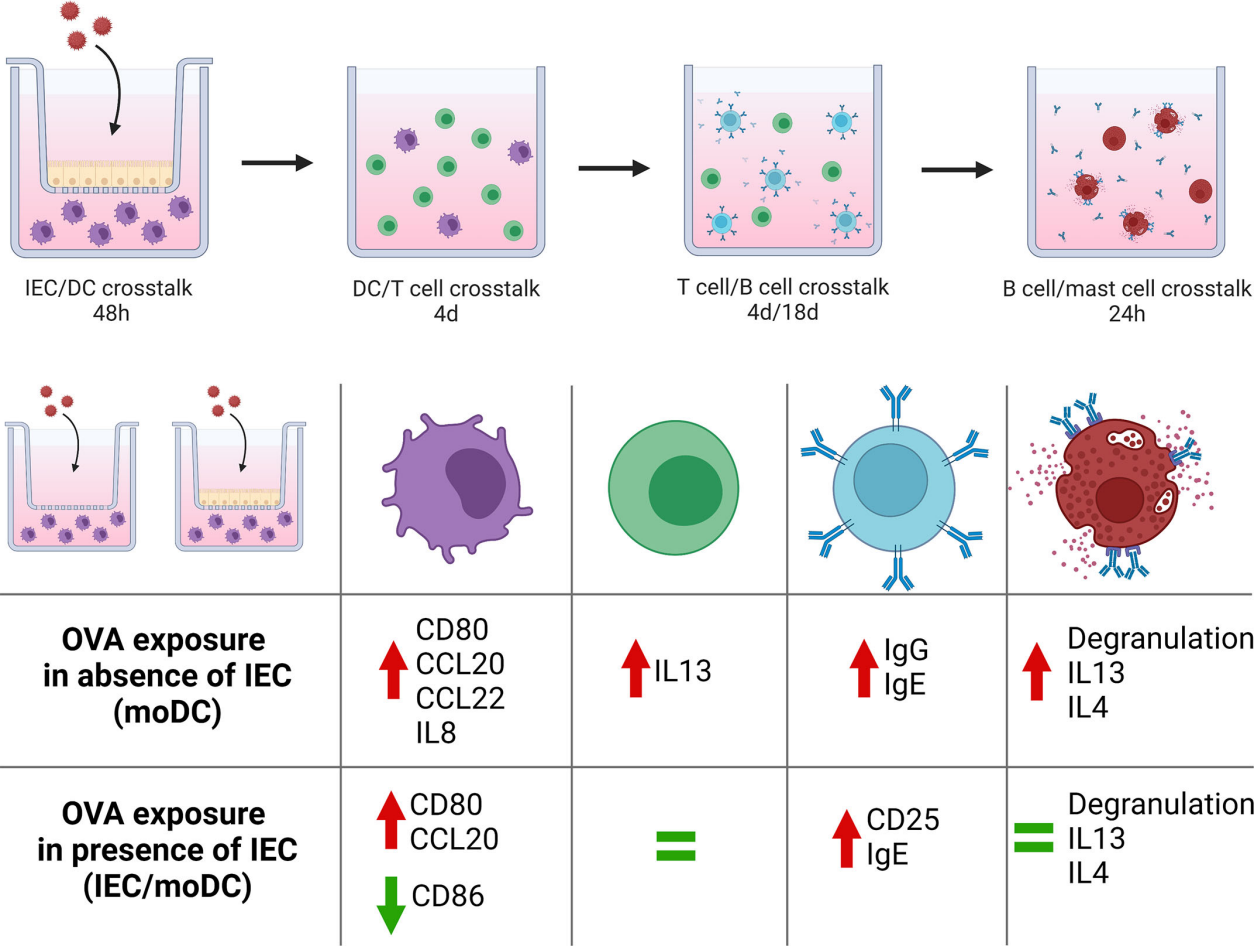
Schematic overview of the coculture steps from our novel sequential mucosal food sensitizing allergenicity model and findings both in absence and presence of IEC during OVA exposure. OVA exposure in the absence of IEC resulted in mast cell activation downstream in the model, while the presence of IEC during OVA exposure did not result in mast cell activation despite the presence of IgE. This figure was created with BioRender.com.

An increasing number of individuals is suffering from food allergies. With the appearance of novel food proteins in our diets, safety testing to identify proteins with high potential to cause allergic sensitization is becoming more relevant before these products enter the market (1). We aimed to develop the first fully human *in vitro* model to mimic the sequential steps that can lead to allergic sensitization *via* the mucosal immune system including the effector phase. For this approach, the hallmark food allergen ovalbumin was chosen for exposure to moDCs in absence and presence of IECs. Subsequent steps consisted of crosstalk between OVA-primed moDCs and naïve Th cells, followed by a coculture of primed naïve Th cells with naïve B cells to investigate immunoglobulin production and the capability of the T cells to instruct IgE isotype switching. Finally, the effector phase was reproduced by studying mast cell degranulation upon culture with B cell supernatant, completing the series of key events involved in the food allergic sensitization and effector response. Allergen-specific responses are difficult to achieve since they require cells with already developed memory responses to a determined protein as was shown for peanut (16). However, the current model is based on moDCs, naïve T and B cells from healthy donors, which could also be applied for novel introduced proteins. This model shows the generic type 2 driving capacity by food allergenic proteins (sensitizing allergenicity), in this case OVA, to provoke allergic sensitization and effector responses without the requirement of using cells from patients allergic to the protein of interest. An overview of our findings is presented in **Figure 6**.

Exposing the HT-29 cell line, as IEC, to ovalbumin enhanced secretion of type 2 driving alarmins. Contributions of these alarmins in modulating DC function enabling them to drive Th2 cell development have been previously studied (4, 17–19). Murine studies revealed that increased epithelial IL33 secretion is sufficient for *in vivo* DC activation leading to peanut allergy, independent of IL25 and TSLP (17). However, although the authors suggest that IL33 is required to induce upregulation of OX40L in DC, we observed an increased OX40L expression in the absence of increased IL33 secretion in OVA-IEC-DC. CD86 upregulation is involved in allergic sensitization as well (20, 21) and we showed a tendency to increased CD86 expression in response to OVA in absence of IEC and a significant release of Th2-polarizing CCL22 (22) and inflammatory IL8 (10, 23) by the moDCs together with a decrease in regulatory TGFβ. Although increased secretion of alarmins and enhanced expression of OX40L on DC is related to inhibited type 1 instructing IL12p70 release (17), both IL12p70 and also type 1 driving IL15 secretion remained unaltered. The current data show that ovalbumin can provoke type 2 activation of both IEC as well as DC. Previously we confirmed that OVA-IEC could drive functional type 2 differentiation in DC, but in the latter study IEC were exposed to OVA for 24h before the IEC/moDC coculture (13). The current study shows both OVA-IEC-DC as well as OVA-DC to obtain a type 2 moDC phenotype, but their phenotype differs depending on the presence of IEC which are known to shape the innate immune response. The present study uses a relatively high, yet physiological relevant OVA concentration, future studies could focus on exposure to different OVA concentrations and the following mucosal immune responses.

Subsequent coculture of OVA-IEC-DC and OVA-DC with allogenic naïve Th cells demonstrated differential functional polarizing outcomes of these DC. More conventional *in vitro* allergy models mostly do not include IEC (16, 24, 25), while the presence of IEC is important in the initiation of the allergic response (13, 26, 27). Interestingly, OVA-IEC-DC did not alter type 1 or type 2 related cytokine secretion by T-cells, while OVA-DC were capable of driving Th2 polarization as indicated by enhanced IL13 secretion and an increased percentage of IL13-expressing Th cells. Based on the differences in functional immunological outcomes, we hypothesized that even though OVA exposure *via* IEC enhanced CD80 and OX40L expression in OVA-IEC-DC, in these DC CD86 expression was reduced. This may have resulted in a lesser Th2 driving capacity of these DC, while IL-10 secretion was increased (data not shown). Interestingly, the expression of CD86 can be suppressed by epithelial-derived factors resulting in more tolerogenic T cell effects (28). Thus the initial presence of IEC during allergen exposure may have facilitated not only the release of type 2 activating mediators but also regulatory factors that suppressed full DC activation in this model. Yet, previous *in vitro* studies demonstrated a type 2 instructing effect from epithelial cells, which can be due to differences in model conditions (11–13). However, the current study shows a stronger type 2 response to OVA in absence of IEC, while OVA-IEC-DC contribute to enhanced regulatory IL-10 secretion during coculture with T cells as indicator for a more tolerogenic response to the allergen. Furthermore, in a previous study we investigated the contribution of OVA pre-exposed IEC in type 2 development *via* instructing moDC (13). In the current study we use the same OVA source which contains some contamination with endotoxins. In spite of this, the OVA exposed moDC were capable of driving type 2 responses both at the level of T cells and mast cells comparable to DC2 (as shown in **Supplemental Figure 3**). Future studies should therefore further look into the possible contribution of endotoxins in the process of OVA induced allergic sensitization, which may also be applicable under physiologic conditions in mucosal tissues of the intestine.

A Th2-dominant environment is essential to trigger IgE production in B cells. B cells were stimulated with anti-IgM to induce aggregation of the BCR (29) as surrogate signal otherwise provoked by an allergen. The OVA-DC-T cells, which showed phenotypical Th2 polarization, tended to enhance IgE secretion by B cells and the B cell supernatant was leading to anti-IgE provoked primary human mast cell degranulation, as well as both IL-13 and IL4 release by these mast cells. The OVA-IEC-DC-T cells did not induce Th2 polarization, but coculture of these T-cells with B cells enhanced CD25 expression in these B cells, indicating activation. In addition, IgE secretion was significantly increased in OVA-IEC-DC-T-B cells. Therefore, even though these OVA-IEC-DC -T cells did not show typical Th2 polarization, which is generally considered to be necessary for IgE isotype switching (30), these cells were capable of inducing IgE secretion in B cells. Some studies involving human B cells reported that IL21 produced by follicular Th cells also promotes IgE isotype-switching (31, 32), more recently this IgE boosting effect was attributed to a balance between IL21 and IL4, and strong stimulation *via* CD40 (33). However, in contrast with the OVA-DC-T-B cell supernatant, the IgE present in the IEC-OVA-DC-T-B cell supernatant did not lead to mast cell degranulation upon anti-IgE crosslinking, not IL-13 and IL-4 secretion. This sequential model to identify the food sensitizing allergenicity risk has the advantage that also this last effector step is implemented. The model indicates if a food protein is capable of instructing DC to induce T-cell activation, contributing to a humoral response in B-cells (IgE isotype switch)m which is functionally capable of eliciting an IgE mediated effector response. Indeed, beyond IgE, B cells may produce other humoral or regulatory mediators such as IL-10 and TGFβ, which can prevent mast cell degranulation (34, 35). Furthermore, both binding of IgE and IgG to the FcϵRI and several types of FcγR respectively or altered immunoglobulin glycosylation, may provide inhibitory signals affecting the anti-IgE induced mast cell degranulation (36, 37). Therefore, future studies should focus on elucidating the role of regulatory mediators such as IL10 and TGFβ from T and B cells on IgE-mediated mast cell degranulation. This may contribute to the predictive value of the model when studying food sensitizing allergenicity risk.

The OVA-DC-T-B cell supernatant did increase anti-IgE induced mast cell degranulation as well as IL13 and IL4 secretion. Indeed, degranulation and type 2 mediator release by mast cells are the consequences of IgE induced mast cell activation and known to contribute not only to allergic symptom development, but also to further drive allergy development (37). Here, ovalbumin exposure to moDCs in absence of IEC was capable of fully driving sequential type 2 sensitization even facilitating IgE mediated mast cell activation, while the latter was prevented when DC function was modulated by IEC (**Figure 6**).

Although several *in vitro* models to study the development of food allergy have been published (11, 12, 38), to our knowledge this is the first method to fully describe the sequential steps from allergic sensitization towards effector cell activation in a novel developed human *in vitro* model. Here, healthy donors were used and an allogenic DC-T cell interaction was performed, effects from sex and age differences between donors were not taken into account. Future studies should focus on investigating immunological outcomes from different concentration of OVA, different allergens as well as further refinement using immune cells from allergic donors, which may allow analysis of allergen specific immune activation in an autologous moDC-T cell coculture setting (16). Furthermore, further characterization of the immune polarization should be investigated, e.g. the development of Th17 and Tfh cells. Basophils and innate lymphoid cells are also involved in early steps of allergic sensitization and implementing these cells or their mediators may have added value. As curative treatments for food allergic disorders are not available, novel preventive strategies could be studied in this model as well. In addition, this method could be used as a starting point to develop similar models for different mucosal and/or barrier sites, such as the lungs and skin, which are continuously controlling the balance between establishing immunity or tolerance when exposed to immune activating components.

## 5 Conclusion

The introduction of novel food products provides a demand for validated human *in vitro* assays which mimic the mucosal immune system allowing to study the sensitizing capacities of novel food proteins. We introduced a sequential mucosal food sensitizing allergenicity model using ovalbumin to provoke epithelial cell and DC activation, mimicking key events of the food allergic sensitization and effector response. This method demonstrated that ovalbumin-induced mucosal immune activation *via* the epithelium results in downstream IgE production, but not in mast cell degranulation. Direct exposure of moDCs to ovalbumin drives a Th2 and B cell activation, facilitating IgE mediated mast cell degranulation and cytokine release. These opposing effects indicate both an activating as well as a tolerogenic role for the intestinal epithelium in response to food allergen ovalbumin. This *in vitro* model combines multiple key events involved in allergic sensitization which can be applied to study mechanisms in allergy development and the sensitizing allergenicity of proteins.

## Data availability statement

The raw data supporting the conclusions of this article will be made available by the authors upon request.

## Author contributions

Study was designed by MZ and LW. Data collection was performed by MZ and CB-D. MZ and LW analyzed and interpreted data. Manuscript was drafted by MZ and critically reviewed by CB-D, FR, GF, JG, BL, and LW. All authors listed have approved for publication.
