# Supplementary material for: An advanced in vitro human mucosal immune model to predict food sensitizing allergenicity risk: A proof of concept using ovalbumin as model allergen
Source: Front Immunol. 2023 Jan 9;13:1073034. doi: 10.3389/fimmu.2022.1073034 (PMC9869142; doi:10.3389/fimmu.2022.1073034)
Supplement: Supplementary file 1 [file DataSheet_1.pdf]

Supplementary Material

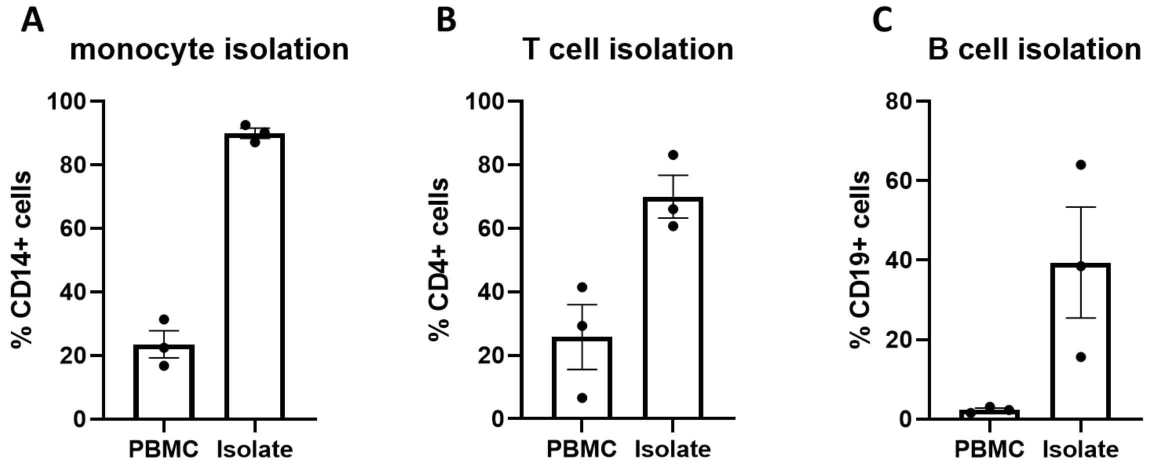

Supplemental Figure 1. Immediately after isolation, purity of A) monocytes, B) naïve T cells and C) naïve B cells was assessed by flow cytometry using the markers CD14, CD4 and CD19 respectively.

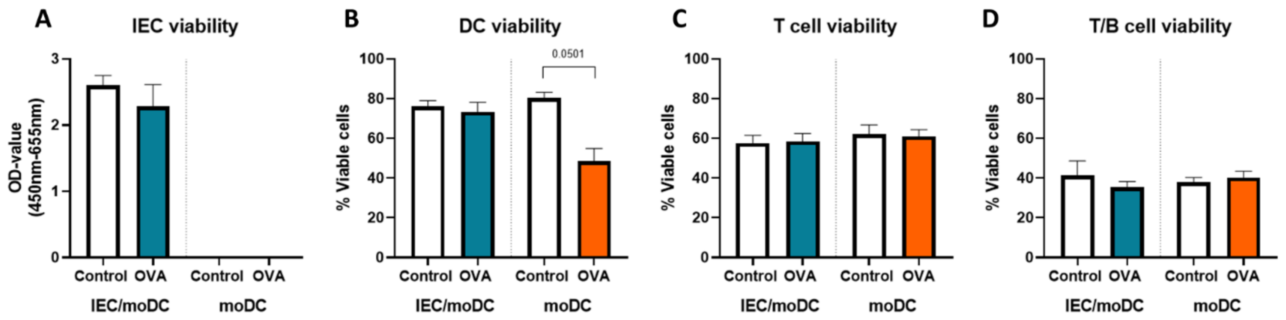

Supplemental Figure 2 Viability of the cells was assessed after each coculture step. A) Mitochondrial activity of the IEC was determined by WST-assay. Viability of B) DCs, C) DC-T cell and D) T cell–B cell coculture was assessed by flow cytometry.

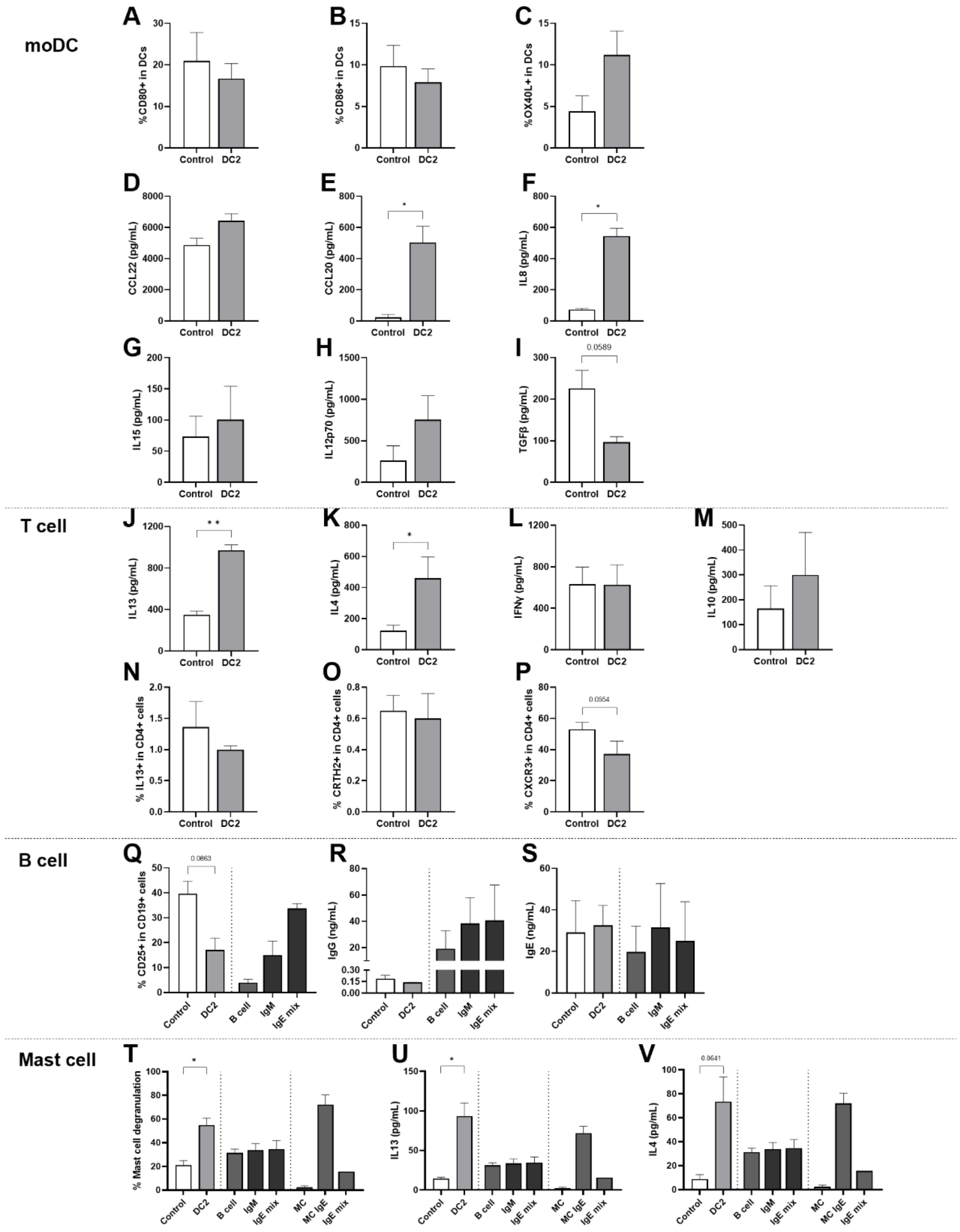

Supplemental Figure 3 Overview of the control conditions used in the sequential mucosal food allergy model. A DC2 driving cytokine mix (consisting of 50ng/mL TNFα, 25ng/mL IL1β, 10ng/mL IL6 and 1µg/mL prostaglandin E2) was applied for 48h to the moDCs to induce maturation into type 2 DCs. Expression of the costimulatory markers A) CD80, B) CD86, and C) OX40L was determined by flow cytometry as well as secretion of D) CCL22, E) CCL20, F) IL8, G) IL15, H) IL12p70, and I) TGFβ in the collected supernatant. After sequential coculture of moDCs with naïve T cells, secretion of K) IL13, L) IL4, L) IFNγ, and M) IL10 was measured in the collected supernatant. Furthermore, T cell expression of N) IL13, O) CRTH2 and P) CXCR3 was assessed by flow cytometry. Following the succeeding coculture of primed T cells with naïve B cells, the activation status of B cells within this T cell/B cell coculture was measured after 4 days as indicated by expression of Q) CD25. In

figure Q, R and S additional controls are included (black bars), these are controls for B-cell responsiveness (not cocultured with T-cells) and composed of non-activated B cells (B cell), anti-IgM activated B cells (IgM) and B-cells exposed to an IgE isotype switching mix (IgE-mix, consisting of 5μg/mL anti-IgM, 5ng/mL anti-CD40 and 20 ng/mL IL4). After 18 days of (T cell/)B cell (co)culture secretion of R) IgG and S) IgE was measured in the collected supernatant. Next, the collected supernatant from the B cell experiments was incubated for 24h with primary human mast cells, after which T) the percentage of mast cell degranulation was calculated upon anti-IgE incubation, and following another 18h overnight incubation, secretion of mast cell derived U) IL13 and V) IL4 was quantified. In figure T,U and V, the last three bars (dark grey) represent controls for the mast cell responsiveness with non-stimulated mast cells as negative control (MC), IgE-exposed mast cells were used as positive control (MC IgE) and IgE mix (5μg/mL anti-IgM, 5ng/mL anti-CD40 and 20 ng/mL IL4) stimulated mast cells were used to control for non-IgE mediated mast cell responses (IgE mix) since the IgE mix B-cell supernatant that was added to the mast cells also contained these stimuli. These last three MC control conditions were not overnight incubated with supernatants derived from (T/)B cells. Control and DC2 conditions are analyzed by paired t-test, n=3, mean ± SEM (* $p<0.5$, ** $p<0.01$).

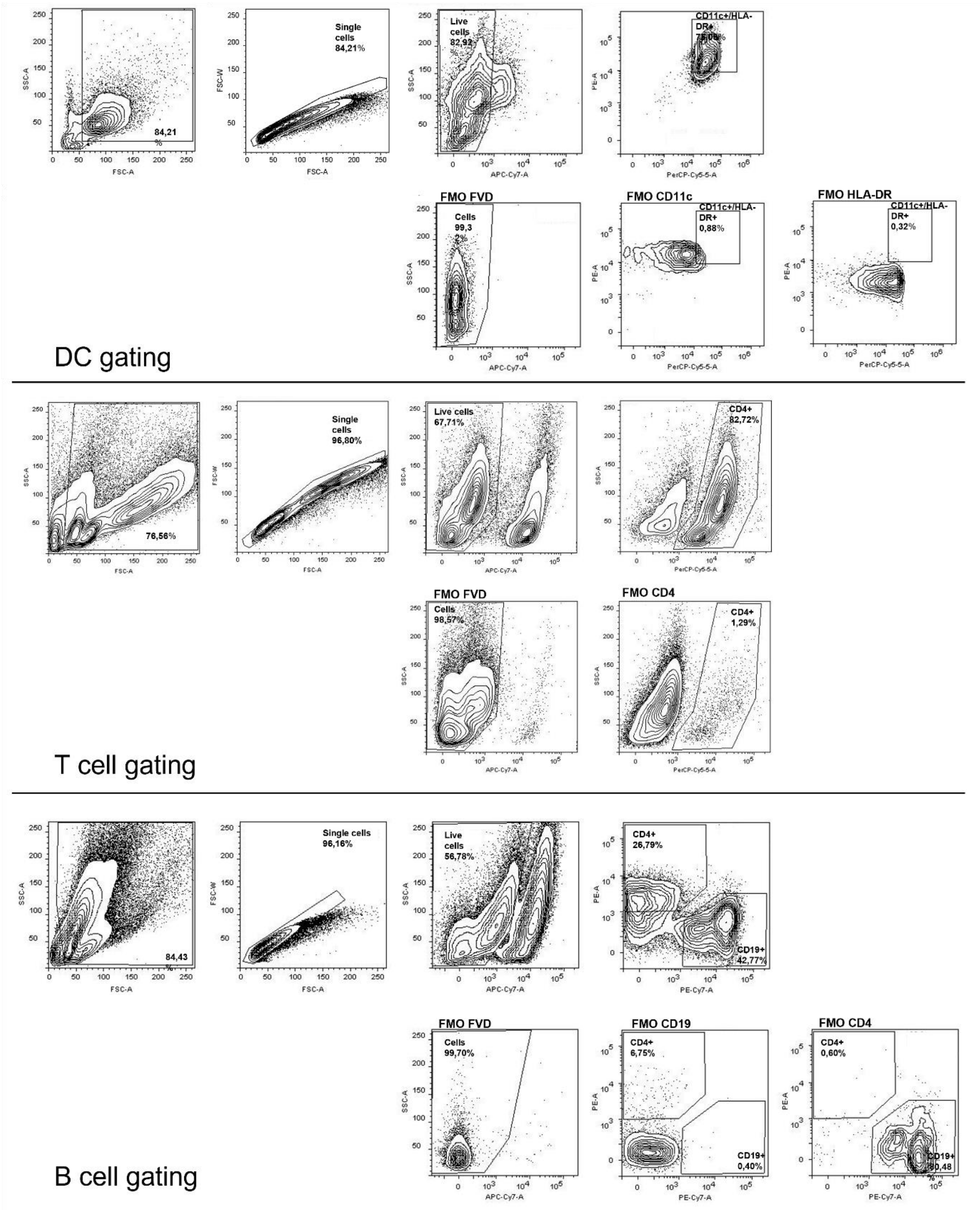

Supplemental Figure 4 On top the gating strategy to determine DC populations, in the middle the gating strategy to determine T cell populations and on the bottom the gating strategy to determine B cell populations are shown using representative samples and corresponding FMO controls.

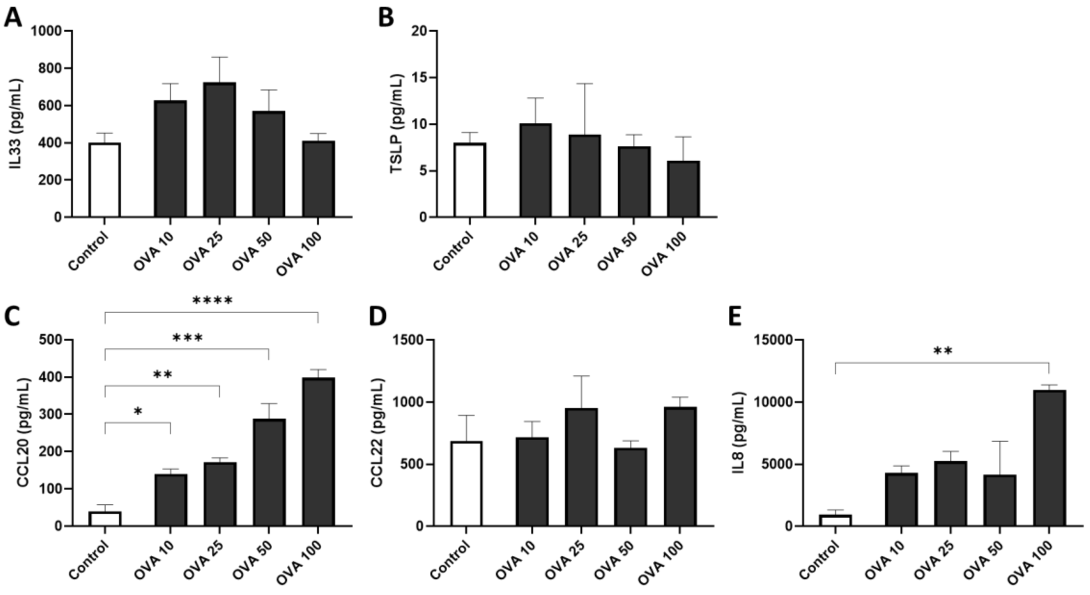

Supplemental Figure 5 Optimal dose of OVA was determined by exposing 3 different passages of IEC to a dose-response in 96 wells plate. Confluent HT29 cells were exposed for 48h to 10, 25, 50 or 100μg/ml OVA in 200μL medium. After 48h, the supernatant was collected and stored for cytokine analysis by ELISA. The levels of A) IL33 and B) TSLP were unaffected by any of the OVA concentrations. C) CCL20 levels followed a dose-dependent significant increase, which was not observed for D) CCL22. Secretion of E) IL8 was only significantly enhanced after exposure to 100μg/ml OVA. Based on these findings, following experiments were conducted with 100 μg/ml OVA. Data is analyzed by One-Way ANOVA and Dunnett posthoc test, n=3, mean ± SEM (* $p<0.05$, ** $p<0.01$, *** $p<0.001$, **** $p<0.0001$).
